# Biological maturity vs. relative age: Independent impact on physical performance in male and female youth handball players

**DOI:** 10.5114/biolsport.2024.132999

**Published:** 2023-12-20

**Authors:** Alfonso de la Rubia, Adam Leight Kelly, Jorge García-González, Jorge Lorenzo, Daniel Mon-López, Sergio Maroto-Izquierdo

**Affiliations:** 1 Deporte y Entrenamiento Research Group, Departamento de Deportes, Facultad de Ciencias de la Actividad Física y del Deporte, Universidad Politécnica de Madrid, Madrid, Calle Martín Fierro, 7. 28040, Spain; 2 Research Centre for Life and Sport Sciences (CLaSS), School of Health Sciences, Department of Sport and Exercise, Birmingham City University, Birmingham, West Midlands, UK; 3 Departamento de Ciencias Sociales, Facultad de Ciencias de la Actividad Física y del Deporte, Universidad Politécnica de Madrid, Madrid, Calle Martín Fierro, 7. 28040, Spain; 4 i+HeATLH, Strategic Research Group, Deparment of Health Sciences, European University Miguel de Cervantes. Valladolid, Calle del Padre Julio Chevalier, 2, 47012, Spain

**Keywords:** Talent development, Talent identification, Team sport, Maturation, Performance, Physical training

## Abstract

Maturity status and relative age are two of the determining factors in talent development. The aim of the study was to analyze the influence of biological maturity status and relative age on physical performance in young male and female handball players. The sample included 48 males (14.11 ± 1.17 years) and 41 females (14.25 ± 1.64 years) players from one Spanish professional handball academy. Anthropometric data (height, sitting height, body mass and self-reported biological parent heights) and physical performance data (CMJ, DJ, 20 m speed, T-test and throwing velocity) were collected. Biological maturity status was determined as the percentage of predicted adult height, while relative age was estimated in birth quartiles based on biennial age grouping (Q1–Q8). The results showed a positive correlation between maturity status and CMJ in male players (*p* < 0.01). Differences in CMJ performance according to maturity status were identified (*p* < 0.05), with higher jump heights being recorded especially in early maturing boys (*p* < 0.01) and first lines and wings (*p* < 0.05). The variance in CMJ test scores could be explained by the maturity status by 42.90% in U–15 (*p* < 0.05) and 72.60% in U–16 male players (*p* < 0.001). By contrast, no differences were found in girls (*p* > 0.05). Moreover, no relationships were found between relative age and indices of physical performance (*p* > 0.05). Overall, maturity status had greater impacts on the tests of physical performance than relative age. Stakeholders should monitor the maturity status of young handball players to avoid physical performance biases that do not allow them to develop their sporting potential.

## INTRODUCTION

Recruiting players for talent pathways with the aim of developing them towards high performance is one of the main purposes of handball academies between the ages of 7–8 and 15–16 years [1]. Young talents often enjoy a wide range of high quality sporting experiences with a positive impact on both the player (e.g., increased technical-tactical level) and the club (e.g., reputational gain) [2]. However, there are some influential factors in the talent development process that may modify the transition from the handball academy to high performance contexts, such as relative age and maturity status [3–5]. Whilst relative age and maturity status are two different constructs that are often used interchangeably, they are easily confused in the talent identification and development systems implemented by team sports academies [6].

The difference in chronological age between athletes of an age group according to their birthdate is known as ‘relative age’ [7]. This concept reflects the age difference between athletes born in the same selection year or biennial cycle, delimited by a previously established cut-off date, and its consequences are known as ‘Relative Age Effects’ (RAE or RAEs) [8]. This phenomenon has been analysed in different handball contexts with different results [9, 10]. However, the majority of research has identified a higher prevalence of RAEs in males and in formative categories (age groups 10/12–16/18 years), attenuating as players get older and with some variance relative to playing position or the demands of the sport [11].

Biological maturation is defined as the rate of progress towards adulthood and can be considered through the concepts of ‘status’, ‘timing’, and ‘tempo’ [12]. The ‘when’ and at what rate maturational development occurs are factors that differ between young players, especially between the ages of 11 to 16 years for boys and 10 to 15 years for girls [13, 14] with the potential for substantial variance in age of peak high velocity (i.e., some children maturing well in advance or delay of their same age peers). Therefore, the maturity status of young players of the same chronological age could differ considerably, as Johnson [14] demonstrated by identifying a five-to-six-year differential between athletes of the same age group in relation to skeletal age and somatic maturity. Individual differences in maturity timing are largely governed by a combination of genetic and environmental factors, and are of particular relevance from early adolescence when pubertal changes in size and athleticism can afford specific (dis)advantages [15].

Individual differences in growth and maturation are critical for the identification and development of talented young athletes. Thus, early maturational timing increases the likelihood of selection for talent programmes in sport compared to peers in the same age group with later maturation [4]. For example, Johnson et al. [16] identified that early maturing football players were more likely to be selected into England’s elite academies as the player progressed through the different age groups and, consequently, selection bias increased. Furthermore, this same study reported that players with a more advanced maturity status tended to be retained in the system around 20 times more than on-time and late maturers. One of the main reasons for this is the relevance of anthropometric and physical factors in performance. Thus, on-time or late maturers tend to be less tall and have less lean muscle mass [17], perform lower on strength and power tests [18], and develop 20% lower sprint speed than early maturers [19].

Considering the impact of the RAE on talent identification and development programmes, it is worth asking whether maturity status and relative age converge and whether one of them is more prevalent throughout the process, especially associated with physical performance. A study by Parr et al. [5] concluded, with a sample of 84 football players aged 11–16 years, that the mixed impact of relative age and maturity status on physical performance was *differential* but *small*, not considering both factors mutually influential. Moreover, separate analysis yielded different correlation values between physical performance and RAEs (*r* = 0.19 – 0.23; *weak*) and maturity status (*r* = 0.71 – 0.75; *strong*). Cumming et al. [20] and Sweeney et al. [3] went one step further affirming that relative age should not be treated as a proxy indicator for biological maturation because older age within an age group would not necessarily imply more advanced maturation. In the same line, Hill et al. [21] and Johnson et al. [16] argued, not only that RAE and maturity status operate separately, but that there is a time lag in their onset and evolution. Thus, while RAEs appear in late childhood and persist into adolescence and decline with age, differences with regard to maturity status do not emerge until puberty and increase with age. For example, Radnor et al. [4] found in a study of young English foot-ballers that 33% of players born in Q4, although fewer in absolute terms, had early maturity status compared to Q1, Q2 and Q3 (10–30%).

In women’ sport, the impact of maturity status and relative age on physical performance is less clear-cut. Girls’ physical qualities (e.g., strength), although they develop earlier than boys’ due to earlier maturation, do not increase as markedly throughout the sport development process as boys’ [15]. Furthermore, early maturation (i.e., increased body size and/or fat mass levels) may be detrimental in sports with high agility and coordination requirements [17]. These differences could influence performance in sports where physical attributes are decisive, such as handball [22]. With regard to RAE, there is no homogeneity of results. [23]. Factors such as the depth of competition, popularity or the number of active participants could be determinants for the lack of impact of relative age on player recruitment processes and competition performance in women’ sport [24].

A number of studies in different sports have considered the relationships between relative age, maturity status, and physical performance in young male athletes [25, 26]. To the best of our knowledge, no study in handball incorporating the female gender has been carried out. Moreover, this phenomenon is yet to be explored in the context of handball, which is a sport characterised by physical attributes and thus leaves young athletes vulnerable to the aforementioned biases. Therefore, the aim of the present study was to analyze the influence of biological maturity status and relative age on physical performance in young male and female handball players. According to previous research, the hypothesis of this study was that the biological maturity status of Spanish handball academy players had a greater impact than relative age on physical fitness tests performed.

## MATERIALS AND METHODS

### Sample

A total of 89 handball players (males: *n* = 48; females: *n* = 41) belonging to U–13 (*n* = 30), U–14 (*n* = 17), U–15 (*n* = 18), U–16 (*n* = 15) and U–17 (*n* = 9) age groups of the Spanish Handball Academy volunteered to participate in the cross-sectional study. According to playing position, the players were distributed as goalkeepers (*n* = 7), wings (*n* = 30), first lines (center backs and backs) (*n* = 35), and pivots (*n* = 17). The inclusion criteria comprised of belonging to the structured training programme implemented by the academy’s qualified coaches whose development starts between the ages of 6 and 8 years in the club’s schools. Furthermore, none of the players reported being injured at the testing time or having suffered a disabling injury or pathology in the previous three months. The parents and/or legal guardians of the minor players were duly informed by means of an informed consent in order to be able to extract, record, and use the data derived from the study. The project and the scientific use of the data was approved by the Ethics Committee of the Universidad Politécnica de Madrid (2020-089; 2020-090; 2020-091) in compliance with the Declaration of Helsinki.

### Procedures

The physical performance tests were conducted based on the recommendations established by the National Strength and Conditioning Association (NSCA) [27], with all players at rest before completing the tests in the following order: (a) anthropometric measurements, (b) physical fitness tests, and (c) sport-specific tests. Anthropometric data were used to calculate the biological maturity status.

### Anthropometric Assessment

Standing height was measured with a stadiometer to the nearest 0.1 cm. (SECA, 216, Vogel & Halke, Hamburg, Germany). In addition, a 40 × 50 × 30 cm wooden anthropometric box (Smart Met, Jalisco, Mexico) was used for sitting height. Body mass was measured to the nearest 0.1 kg. on a digital scale (SECA, 876, Vogel & Halke, Hamburg, Germany). Parental stature was provided in centimetres for each father and mother and then converted to inches to adjust for overestimation [28]. The following adjustment factor was used for males (*y* = 2.316 + 0.955*x*) and for females (*y* = 2.803 + 0.953*x*), where ‘*y*’ was the adjusted height and ‘*x*’ was the self-reported value. All the measurements were carried out according to the International Standards for Anthropometric Assessment (ISAK) [29] by accredited staff (level 2).

### Biological Maturity Status

The biological maturity status was calculated from the Khamis-Roche equation validated especially for white Caucasian children. This equation provides the predicted adult height (PAH) using a regression formula based on gender-specific coefficients [30]. Estimated biological maturity status was expressed as a z-score relative to age-specific reference values for males and females, using the percentage of predicted adult height (% PAH) attained at the time of measurement and half-year age and sex-specific means and standard deviations from the Berkeley Guidance Study [31]. These maturity status classifications have shown a moderate correlation with other maturational classifications based on skeletal age in both males and females [32]. The z-scores associated with the % PAH were used to classify handball players as “late maturers”(z-score < – 0.5), “on-time maturers” (-0.5 < z-score < + 0.5), or “early maturers” (z-score > + 0.5), as in other studies of youth team players. This less conservative and more sensitive criterion was used in order to reduce the differences derived from categorising players according to notably different maturity status, as with the traditional criterion (± 0.99 z-score) we could find a larger differential in growth and maturation determinants factors such as skeletal age (approximately two years) [21, 33].

### Relative Age

Players were categorised into eight quartiles (Q) according to relative age based on their birthdate and cut-off date for the respective biennial cycle established by the International Handball Federation (IHF). The players were grouped as follows according to even/odd-numbered years: Quartile 1/5 (Q1/Q5): January 1–March 31; Quartile 2/6 (Q2/ Q6): April 1–June 30; Quartile 3/7 (Q3/Q7): July 1–September 30; Quartile 4/8 (Q4/Q8): October 1–December 31.

### Physical Fitness Tests

The countermovement jump (CMJ) and drop jump (DJ) test were performed to evaluate the elastic-explosive manifestation and reactive strength of the lower body strength respectively. The tests were carried out on a contact platform (Chrono Jump Bosco System ®, Spain) and the jump height was measured in centimetres. The margin of error of the microcontroller was 0.1% and the validity of the fiberglass platform was 0.95 (ICC) [34]. Prior to the test, the players were instructed in each of the jumping technique [35]. Both tests were preceded by a warm-up jump at 50% and 75% of the self-determined maximum effort of the players and each of them performed a practice jump for familiarisation. Three attempts were made with a 60-seconds passive recovery [36]. If the range of jump height variation was ^3^ 0.4 cm, additional jumps were performed until the threshold was satisfied and the mean height was taken. The difference in variances between groups (two vs. three attempts) was tested by means of a t-test with a significance level of *p* > 0.05.

The T-test was conducted to examine the timed agility of the players according to Pauole et al. [37] protocol. Players were instructed to finish the ‘T’ circuit (9.14 m × 4.47 m on both sides) in the shortest time. Prior to test, all players completed warm-up efforts at 75% of their self-determined maximum and were allowed to perform a familiarisation attempt with the circuit. Two attempts were made, with a 2-minute passive recovery between them, taking the average time of both as the final test result measured by a photoelectric cell gate (Chrono Jump, Bosco System, Barcelona, Spain) with almost perfect reliability (*ICC* = 0.999–1.000) [34].

The 20 m linear speed test was used to evaluate the running speed of players. The course will consist of a straight-line run asking the players to run at the maximum possible speed to cross two photoelectric cell gates (Chrono Jump, Bosco System, Barcelona, Spain; *ICC* = 0.999–1.000) [34] separated by 20 m. Time and speed were recorded after the completion of two attempts separated by a 3-minute passive recovery, having previously warmed up to 75% of their maximum self-perceived effort. The best 20 m time was used for further analysis.

The throwing speed test was performed to measure the speed in the specific throwing action in handball. Thus, the speed was examined in a throwing situation at a distance of 9 m with a 3-step run and a final jump with one foot. The ball used was the one established by the IHF regulations for each gender and competitive level. A radar gun (Stalker Pro Inc., Plano, TX, USA; *ICC* = 0.987–1.000 [38]), with a sampling frequency of 100 Hz and a sensitivity of 0.045 m/s, was placed 1 m behind the goal net and perpendicular to the player with the aim to eliminate possible angle errors [39]. Prior to the test, a standardised warm-up specific to handball was performed. Each player made two attempts with a 1-minute passive recovery. The best speed result was used for further analysis.

### Statistical Analysis

The analyses were conducted with SPSS (Version 26). Data are expressed as mean and standard deviation (*X ± SD*). Absolute and relative frequency counts were used to determine the number of handball players within each quartile (Q1–Q8) and each biological maturity z-score (i.e., early, on-time, late), according to gender, playing position, and age group. The assumption of normality of each variable was assessed by Kolmogorov–Smirnov test (n ^3^ 50), and Shapiro-Wilk’s test (n < 50) and Levene’s test was carried out to verify the homogeneity of variances. A Pearson’s correlation was run to analyse the relationship between biological maturity status and relative age and the physical fitness tests. A one-way ANOVA was conducted to test for differences in physical fitness tests between groups according to the biological maturity status and relative age. Moreover, Tukey-Kramer’s post hoc test was performed to confirm the difference between biological maturity groups. Partial eta-squared (*ŋp*^2^) was used to evaluate the effect size of the differences between groups, considering small (0.01), medium (0.06), or large value (0.14) [40]. Furthermore, a linear regression was performed to examine the impact of biological maturity status and relative age on physical fitness tests scores. The value of *p* < 0.05 was set as significant for all statistical comparisons.

## RESULTS

**FIG. 1:**
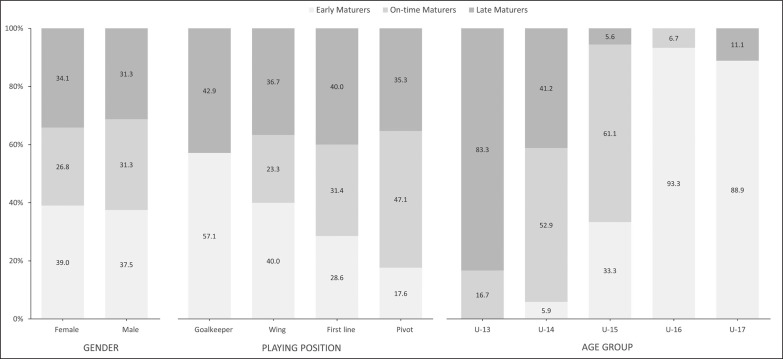
Player characteristics by biological maturity status.

The sample descriptive statistics according to the biological maturity status and relative age of male and female players are shown in Figures 1 and 2(a-c), respectively. With regard to maturity status, 34 early (38.20%), 26 on-time (29.21%), and 29 late maturers (32.59%) were identified. In relation to relative age, the sample was distributed in the following quartiles (Q): Q1 (n = 10; 11.3%); Q2 (n = 8; 9.00 %); Q3 (n = 6; 6.70 %); Q4 (n = 8; 8.90 %); Q5 (n = 11; 12.30 %); Q6 (n = 21; 23.60 %); Q7 (n = 15; 16.90 %); and, Q8 (n = 10; 11.20 %).

**FIG. 2a:**
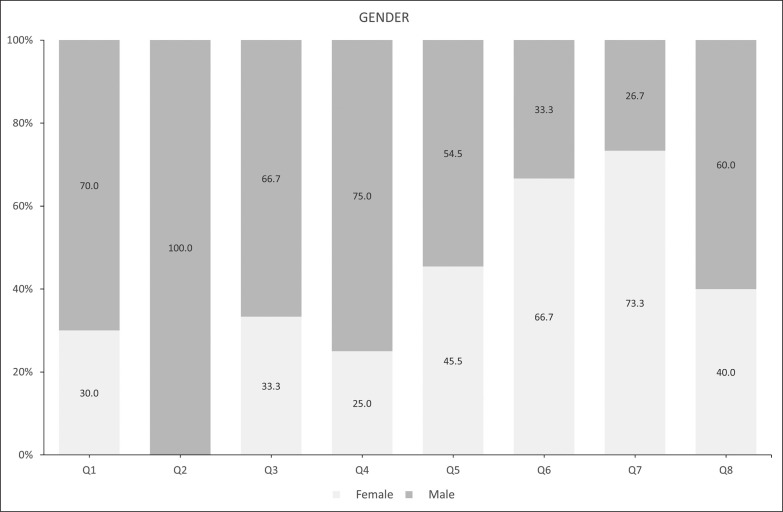
Player characteristics by relative age according to gender.

**FIG. 2b:**
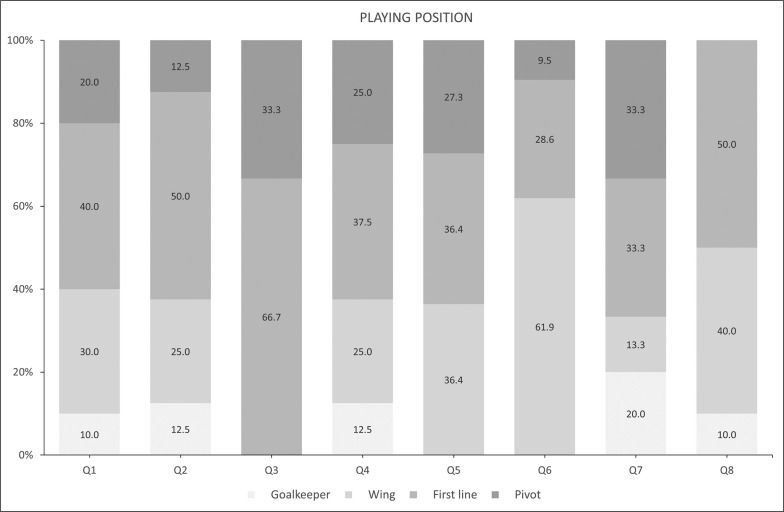
Player characteristics by relative age according to playing position.

**FIG. 2c:**
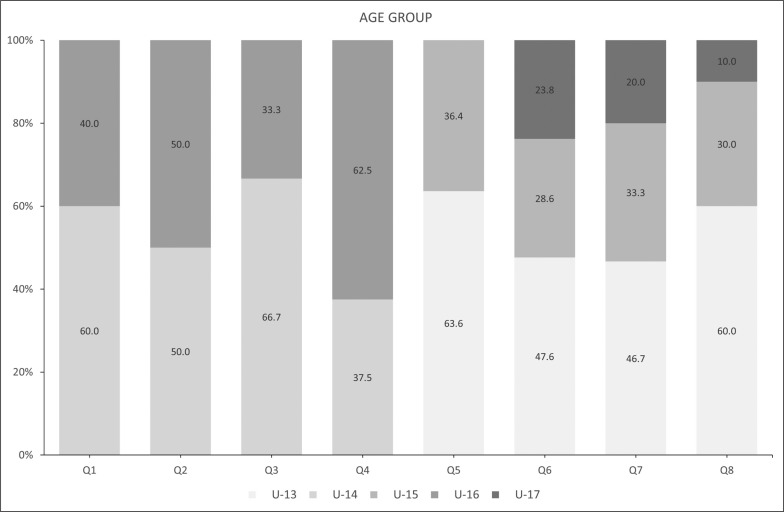
Player characteristics by relative age according to age group.

**FIG. 3:**
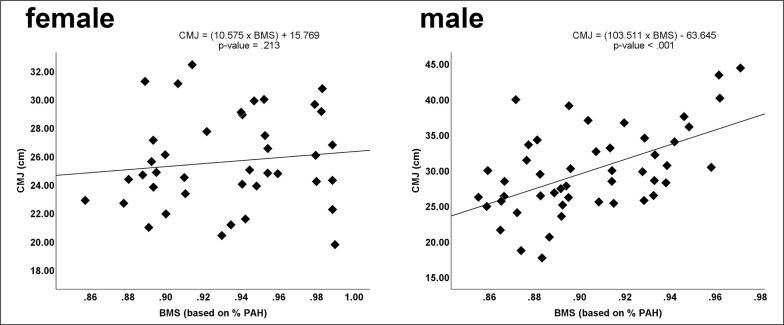
CMJ test scores of male and female handball players according to biological maturity status (BMS) based on percentage of adult height (% PAH).

The correlations between biological maturity status and relative age and the five physical fitness tests are presented in Table 1. A positive correlation between biological maturity status and CMJ (*r* = 0.540; *p* < 0.001) was identified in male players. Figure 3 presents in detail (scatter plot) the marks obtained by male and female players in the CMJ test according to the biological maturity status based on the percentage of adult height (% PAH).No significant correlations in the remaining physical fitness tests with regard to the biological maturity status and relative age were observed (*p* > 0.05, all).

**TABLE 1:** Correlation between relative age/biological maturity status and physical fitness tests according to gender.

		Physical Fitness Tests				

		CMJ	DJ	20 m. speed	T-test	Throwing velocity
Women Players (n = 41)	RA	0.20	−0.15	0.18	−0.04	0.09
	BMS	0.12	0.01	0.08	0.01	0.11

Men Players (n = 48)	RA	−0.17	−0.21	−0.05	0.02	0.14
	BMS	0.54^**^	0.23	0.01	0.05	−0.13

All Players (n = 89)	RA	−0.19	−0.18	0.08	0.02	0.08
	BMS	0.16	0.10	0.08	0.06	−0.05

Notes: CMJ = countermovement jump; DJ = drop jump.

*p < 0.05;

** *p* < 0.001.

Table 2 shows the performance values (*X ± SD*) for each of the five physical fitness tests performed according to biological maturity status, relative age, and gender. The CMJ test results showed significant differences between the different maturity groups (*F*_(2,86)_ = 4.55, *p* < 0.05, *η*^2^ = 0.10), with higher jumping height values in the early maturers (*X ± SD* = 29.99 ± 5.96) than late maturers (*X ± SD* = 26.07 ± 4.62). Analysing the sample by gender, the impact of the biological maturity status on the CMJ test was significant in boys (*F*_(2,45)_ = 5.72, *p* < 0.01, *η*^2^ = 0.20), with higher jump scores registered in the early maturers (*X ± SD* = 33.53 ± 5.85) than in the late maturers (*X ± SD* = 27.06 ± 5.65), while in girls the effect disappeared (*p* > 0.05). According to playing position, biological maturity status influenced jump height in the CMJ test carried out by first lines (*F*_(2,32)_ = 3.92, *p* < 0.05, *η*^2^ = 0.20), with higher values in early (*X ± SD* = 30.42 ± 5.52) than in late maturers (*X ± SD* = 25.04 ± 3.67). No significant correlation was found in the remaining physical fitness tests (*p* > 0.05). No correlation was also found between relative age and physical fitness tests (*p* > 0.05) (Table 3).

**TABLE 2:** Anthropometric characteristics and descriptive statistics (*X ± SD*) of physical fitness tests according to the biological maturity status (early, on-time and late) of female and male handball players.

	Early Maturers		On-time Maturers		Late Maturers		*p*	*ηp* ^2^

	*X*	*SD*	*X*	*SD*	*X*	*SD*		
Height								
All	167.42	7.81	162.09	7.66	162.38	7.52	-	-
Females	163.69	5.09	158.38	1.63	159.42	3.17	-	-
Males	170.91	8.43	164.80	9.18	165.01	9.23	-	-

Weight								
All	59.44	7.05	55.60	3.61	56.22	5.07	-	-
Females	58.69	4.52	52.31	2.10	52.85	4.37	-	-
Males	60.15	8.91	58.01	2.33	59.22	3.57	-	-

CMJ								
All	29.99^a^	5.96	28.20	4.87	26.07^a^	4.62	0.013^*^	0.10
Females	26.20	3.11	25.85	4.01	24.96	2.87	0.574	0.03
Males	33.53^a^	5.85	29.93	4.84	27.06^a^	5.65	0.006^**^	0.20

DJ								
All	25.56	7.15	21.84	5.33	23.18	8.37	0.154	0.04
Females	24.45	5.27	20.71	6.41	24.12	9.70	0.416	0.05
Males	26.59	8.61	22.66	4.44	22.34	7.18	0.178	0.07

20 m. speed								
All	3.47	0.31	3.46	0.20	3.43	0.28	0.881	0.00
Females	3.50	0.32	3.51	0.18	3.45	0.29	0.841	0.01
Males	3.44	0.31	3.43	0.22	3.42	0.29	0.985	0.00

T-test								
All	12.69	1.25	12.71	0.89	12.55	1.11	0.815	0.01
Females	12.70	1.24	12.92	0.79	12.63	1.19	0.806	0.01
Males	12.68	1.30	12.57	0.96	12.48	1.06	0.873	0.01

Throwing velocity								
All	69.24	9.94	70.67	9.39	70.53	10.65	0.838	0.00
Females	70.24	9.07	67.86	8.90	68.83	11.25	0.834	0.01
Males	68.31	10.93	72.72	9.49	72.04	10.16	0.445	0.04

Notes:CMJ = countermovement jump; DJ = drop jump.

a = differences between early and late maturers;

b = differences between early and on-time maturers;

c = differences between on-time and late maturers

* *p* < 0.05.

** *p* < 0.01.

**TABLE 3:** Descriptive statistics (*X ± SD*) of physical fitness tests according to the relative age (Q1 – Q8) of female and male handball players.

PFT	Q1		Q2		Q3		Q4		Q5		Q6		Q7		Q8		*p*	*ηp* ^2^

	*X*	*SD*	*X*	*SD*	*X*	*SD*	*X*	*SD*	*X*	*SD*	*X*	*SD*	*X*	*SD*	*X*	*SD*		
CMJ																		
All	28.7	7.2	30.6	6.1	27.8	6.9	30.8	6.1	28.0	6.8	26.6	3.8	27.5	4.5	26.5	3.0	0.475	0.08
Females	22.8	2.5	-	-	22.5	3.1	27.0	3.1	26.2	2.8	26.3	3.8	26.2	3.8	24.0	0.9	0.378	0.16
Males	31.3	7.1	30.6	6.1	30.5	6.9	32.1	6.5	29.5	8.9	27.2	4.1	31.1	6.2	28.1	2.8	0.862	0.07

DJ																		
All	26.3	4.8	24.2	7.4	24.1	11.2	24.4	9.4	24.6	9.5	23.5	6.7	21.2	6.1	21.9	5.7	0.802	0.05
Females	27.8	5.8	-	-	19.7	12.3	23.8	9.6	23.7	9.9	24.8	7.6	21.0	6.2	22.3	9.7	0.493	0.08
Males	25.6	4.6	24.2	7.4	26.3	11.8	24.6	10.2	25.3	10.1	20.9	3.6	21.7	6.9	21.6	1.7	0.870	0.07

20 m. speed																		
All	3.3	0.3	3.5	0.3	3.4	0.3	3.5	0.4	3.4	0.2	3.4	0.3	3.6	0.2	3.4	0.3	0.573	0.07
Females	3.2	0.2	-	-	3.6	0.1	3.6	0.6	3.4	0.2	3.5	0.3	3.5	0.2	3.6	0.4	0.633	0.10
Males	3.4	0.3	3.5	0.3	3.3	0.3	3.5	0.4	3.4	0.3	3.4	0.2	3.6	0.5	3.3	0.2	0.599	0.12

T-test																		
All	12.3	1.0	12.7	0.9	12.9	1.3	12.9	1.6	12.9	1.0	12.4	1.2	12.7	0.9	12.7	1.1	0.831	0.04
Females	12.6	0.9	-	-	13.5	0.1	13.1	2.6	12.9	0.7	12.5	1.2	12.6	0.9	13.2	1.4	0.507	0.08
Males	12.2	1.1	12.7	0.9	12.7	1.6	12.8	1.4	12.8	1.3	12.2	1.2	13.1	0.8	12.3	0.8	0.832	0.08

Throwing velocity																		
All	70.6	9.2	68.5	10.6	69.5	11.9	66.6	11.4	70.3	11.0	70.3	11.6	70.6	8.3	73.1	7.3	0.956	0.02
Females	72.0	10.5	-	-	59.2	9.5	72.0	11.3	67.2	5.9	67.4	12.6	71.1	7.8	72.6	7.0	0.644	0.10
Males	70.0	9.4	68.5	10.6	74.6	10.1	64.8	11.9	72.9	14.0	76.0	7.1	69.2	10.8	73.4	8.1	0.613	0.12

Notes: PFT = physical fitness test; CMJ = countermovement jump; DJ = drop jump. ^*^p < 0.05. ^**^p < 0.01.

Physical fitness tests regressions carried out by gender showed biological maturity status in boys explained 27.90% of the variance obtained in the CMJ test (*R*^2^ = 0.28, *F*_(1,46)_ = 19.18, *p* < 0.001), rising to 61.70% in early maturing boys (*R*^2^ = 0.62, *F*_(1,13)_ = 23.54, *p* < 0.001). In girls, biological maturity status explained 31.60% of the variance registered in the CMJ test on-time maturers (*R*^2^ = 0.32, *F*_(1,9)_ = 5.61, *p* < 0.05).

In relation to age group, 19.30% of the change in T-test scores achieved by U–14 players (*R*^2^ = 0.19, *F*_(1,15)_ = 4.83, *p* < 0.05) and 37.40% of the change in CMJ scores performed by U–16 players (*R*^2^ = 0.37, *F*_(1,13)_ = 9.35, *p* < 0.01) were explained by the biological maturity status.

In the combined gender and age group analysis, 38.50% of the variance recorded in the throwing velocity test scores in U–17 female players (*R*^2^ = 0.38, *F*_(1,7)_ = 6.00, *p* < 0.05) and 31.70% of the variance in the CMJ test scores in U–13 female players (*R*^2^ = 0.32, *F*_(1,14)_ = 7.97, *p* < 0.05) were explained by the biological maturity status. In U–15 and U–16 male players, the variance in CMJ test scores could be explained by the biological maturity status by 42.90% (*R*^2^ = 0.43, *F*_(1,7)_ = 7.02, *p* < 0.05) and 72.60% (*R*^2^ = 0.73, *F*_(1,12)_ = 32.79, *p* < 0.001), respectively.

Based on playing position, the biological maturity status explained 29.90% of the CMJ test scores performed by male first line players (*R*^2^ = 0.30, *F*_(1,17)_ = 8.69, *p* < 0.01) and 50.80% of those achieved by male wing players (*R*^2^ = 0.51, *F*_(1,13)_ = 15.47, *p* < 0.01).

Regression analyses showed no differences in physical fitness across the RAE groups (*p* > 0.05).

## DISCUSSION

The present study aimed to analyse the impact of biological maturity status and relative age on physical fitness tests performed by male and female players belonging to a Spanish handball academy based on three modulating variables (i.e., gender, playing position, and age group). Results showed biological maturity status was associated with CMJ performance in male players whereas relative age presented no relationship level with the scores obtained in the physical fitness tests. Specifically, an advanced maturity status was associated with higher performance in the CMJ test, especially in the case of early maturing boys (by gender), belonging to the U–15 and U–16 male age groups (by gender and age group), and first lines and wings (by playing position). On the other hand, no differences in physical performance were found among female players according to the biological maturity status and relative age. Nevertheless, biological maturity status functioned as a performance predictor, less powerful than for boys, in the throwing velocity test in U–17 female players and in the CMJ test in U–13 female players.

The main result indicates physical performance in young hand-ball players was more influenced by biological maturity status than by relative age. Similar results were obtained by Parr et al. [5] in a sample of 84 male football players aged between 11.3 and 16.2 years confirming a higher correlation between biological maturity status and physical performance. Moreover, a recent study in two English football academies with male players aged between 8.1 and 18.9 years found that the impact of maturity status on sprinting ability was greater than that of relative age, with early maturers obtaining better times and velocities [4]. Therefore, this evidence would support the idea that the two constructs–biological maturity status and relative age–operate independently and the functional advantages associated with an advanced maturity status may not be attributed to RAEs [16].

It should be noted that the two factors (biological maturity status and relative age) do not coincide in the same period of children’s development so the physical (dis)advantages on the talent selection process will not be the same. [6]. Thus, while RAEs operate in child-hood and is maintained (although generally decreasing) throughout adolescence, the maturational bias emerges with the onset of puberty and increases in magnitude with adolescence [12, 21]. Indeed, Johnson et al. [16] identified a higher likelihood of selection to elite U–17 teams for early maturers (20 times) than for relatively older players at the start of developmental programmes (2.2 times). The most plausible explanation would lie in the temporary gain in physical and anthropometric qualities attributed to early maturers, with late maturers being excluded to a greater extent throughout the formative stages.

Although the impact of the biological maturity status on physical performance seems to be discrete, the scientific literature agrees that the CMJ is one of the tests most influenced by maturity status [4, 5], with high values of variance being in jump height [41]. Specifically, the results of the present study even explained 61.70% of the variance recorded in the CMJ for early maturing male players. Since the CMJ expresses the power of the lower limbs playing a fundamental role in the jumping actions of handball (e.g., defensive blocking) [22], it is interesting to note that male players of advanced maturity status obtained greater jump heights. A higher percentage of lean mass and larger body size (height and weight), due to a more intense adolescent growth spurt, could be the main determinants of the superior physical performance of early maturers [42]. On the other hand, it is worth noting the lack of impact of biological maturity status on sprinting ability. A training method not primarily aimed at improving sprinting ability in handball due to the lower relevance in the game to the detriment of other abilities (i.e., strength) and higher height and weight values at this age (14.17 ± 1.39 years) than other sports such as football [43], could be two of the explanations. Nevertheless, it would be appropriate to analyse this relationship separately for playing positions with a greater displacement speed (e.g., wings).

Furthermore, the impact of biological maturity status on CMJ was amplified in the U–16 male age group (72.60 % of the variance). Entering adolescence produces a series of body changes that lead to the development of higher strength levels [44]. This appears to be multiplied in children aged 11–15 years [45] as well as in early maturers [17], providing a sporting advantage over less mature peers. Neuromuscular gains due to increasingly higher intensity and volume of training as the chronological age increases could explain the performance differential in the CJM test [46]. Therefore, chronological age and biological maturity status could be playing a dual role on jumping ability of handball players in formative sporting stages. On the other hand, a notable influence of biological maturity status (50.80% of the variance) was identified in the CMJ test in wing male players. The game-specific physical demands cause this playing position to require a higher jumping ability [47]. Nevertheless, and in comparison to other playing positions where the variance in maturity status was not as wide (e.g., pivot), it is necessary to highlight that the performance differences in jumping ability is not only influenced by an advanced maturity status but also by a well-developed jumping ability throughout the training process [48].

The results are not as clear for adolescent women as in previous studies [49]. While jumping ability was influenced by biological maturity status in the U–13 age group (31.70% of the variance), throwing velocity was explained by 38.50% in the U–17 age group. This highlights the different impact of the biological maturity status on physical performance among girls throughout adolescence [33]. Early maturing girls typically experience exponential body size growth in early adolescence that allows them to develop higher strength levels in jumping or sprinting tests. However, these strength levels seem not to be as decisive in tests such as the CMJ or 20 m sprint as this stage develops, due to increases in body fat leading to a loss of agility levels [17]. Conversely, lower limb strength levels become less important in the throwing action throughout adolescence to the detriment of anthropometric perimeters and upper limb strength levels [50]. Therefore, this could mean that the impact of the biological maturity status would be greater in higher age groups such as U-17.

This study is not without its limitations. First, the research was carried out in a single academy at a handball club and with an available sample of boys and girls aged over 12 years, so in terms of statistical testing the sample could be considered small. Second, parental heights for the PAH were self-reported, rather than measured, and subsequently adjusted for overestimation. Third, the equation generally used for the prediction of adult height was validated on Caucasian children and may not be appropriate for the calculation of biological maturity status in children of other ethnicities. Fourth, no reference data (i.e., Spanish growth reference data) was used for the calculation of the z-scores which could influence the categorisation of the players according to the biological maturity status. Finally, the non-use of force plates for the evaluation of the jumping tests (CMJ and SJ) did not allow for a full exploration of the performance strategy (e.g., direct measurement of force applied).

## CONCLUSIONS

This study, considered as an initial exploration of the topic, determined the impact of biological maturity status on the physical performance of young handball players, whilst no effect was observed for relative age. Specifically, the maturity status affected the CMJ test scores, showing that the more mature players (male, U–15/U–16, and wings and first lines) performed better than the rest. The results was not as clear-cut for female handball players. These findings should serve to understand, both for stakeholders and practitioners, that these two constructs can have a different influence on the athlete physical performance. Thus, a late maturing or relatively young player should not be relegated within talent development programmes, even to the point of drop-out, but stakeholders should find the right strategy to retain him/her and develop his/her future potential by providing adapted competitive experiences. Therefore, regular growth and maturation assessment within talent development systems is necessary to monitor and track the physical performance of young male and female handball players. The implementation of training and competition strategies based on biological maturational status (e.g., bio-banding) [51] should be considered in order to reduce the player selection and retention bias according to anthropometric and physical factors. Nevertheless, it is worth noting the need for prospective studies with larger samples to analyse the impact of the maturity status and RAE on selection and performance.
